# Low-adhesion culture selection for human iPS cell-derived cardiomyocytes

**DOI:** 10.1038/s41598-024-60765-5

**Published:** 2024-05-15

**Authors:** Tetsutaro Kikuchi, Katsuhisa Matsuura, Tatsuya Shimizu

**Affiliations:** Institute of Advanced Biomedical Engineering and Science, Tokyo Women’s Medical University (TWIns), 8-1 Kawada-cho, Shinjuku-ku, Tokyo, 162-8666 Japan

**Keywords:** Cardiovascular biology, Stem-cell differentiation

## Abstract

Despite progress in generating cardiomyocytes from pluripotent stem cells, these populations often include non-contractile cells, necessitating cardiomyocyte selection for experimental purpose. This study explores a novel cardiomyocyte enrichment mechanism: low-adhesion culture selection. The cardiac cells derived from human induced pluripotent stem cells were subjected to a coating-free low-adhesion culture using bovine serum albumin and high molecular weight dextran sulfate. This approach effectively increased the population of cardiac troponin T-positive cardiomyocytes. Similar results were obtained with commercially available low-adhesion culture dishes. Subsequently, we accessed the practicality of selection of cardiomyocytes using this phenomenon by comparing it with established methods such as glucose-free culture and selection based on puromycin resistance genes. The cardiomyocytes enriched through low-adhesion culture selection maintained autonomous pulsation and responsiveness to beta-stimuli. Moreover, no significant differences were observed in the expression of genes related to subtype commitment and maturation when compared to other selection methods. In conclusion, cardiomyocytes derived from pluripotent stem cells were more low-adhesion culture resistant than their accompanying non-contractile cells, and low-adhesion culture is an alternative method for selection of pluripotent stem cell-derived cardiomyocytes.

## Introduction

Recent advancements have made it possible to generate cardiomyocytes from pluripotent stem cells such as embryonic stem cells or induced pluripotent stem cells, enabling a wide range of studies utilizing human cardiomyocytes. Notably, the utilization of pluripotent stem cell-derived cardiomyocytes for drug evaluation has sparked significant research into assessing cardiotoxicity, including proarrhythmogenicity^1–7^. Despite progress in generating cardiomyocytes from pluripotent stem cells, these populations often include non-contractile cells, necessitating cardiomyocyte selection for experimental purpose. Ensuring a high level of purity in cardiomyocytes is crucial for testing and research purposes. Consequently, active discussions revolve around methodologies aimed at enhancing the purity of cardiomyocytes.

The bifurcation of the fate of contractile cardiomyocytes and their non-contractile derivatives depends on a subtle stoichiometric relationship of differentiation signals, presenting a significant challenge in consistently generating high-purity cardiomyocytes^8,9^. Moreover, studies have reported variations in differentiation efficiency based on the origin of stem cells^8,10^. To obtain the required level of purity for testing and research, several methods have been proposed to select cardiomyocytes from cell populations. One approach involves genetically modifying the cells to become drug-resistant upon differentiation into cardiomyocytes^11^. Another widely used method is culturing cardiomyocytes in a glucose-free medium supplemented with lactic acid, leveraging their unique energy metabolism^10,12^. Although these selection techniques are undeniably valuable, the challenge lies in finding a balance between purity and yield. In other words, increasing the stringency of selection decreases the yield of cardiomyocytes. Therefore, the search for new selection principles that can complement existing methods remains an ongoing challenge.

In this study, we explored a novel mechanism of cardiomyocyte selection based on the difference in low-adhesion culture resistance, which has not been investigated before. Our previous work involved the development of a coating-free low-adhesion culture using bovine serum albumin (BSA) and high-molecular-weight dextran sulfate (DS)^13^. When this approach was applied to human induced pluripotent stem cell (hiPSC)-derived cardiomyocytes, it was unexpectedly discovered that cardiac troponin T (cTnT)-positive cardiomyocytes exhibited relatively higher low-adhesion culture resistance compared to cTnT-negative cells. Motivated by this observation, we aimed to leverage this property to device a selection method and subsequently compared it with other existing selection methods.

Low adhesion culture successfully enriched cardiomyocytes, and those cells exhibited autonomous pulsation and beta-stimulant response comparable to those treated with other selection methods. Furthermore, no significant differences were observed in gene expression concerting subtype commitment or maturation.

## Results

### DS and BSA supplementation blocked cell adhesion and enriched cTnT^+^ cells in serum-free medium

**Figure 1:**
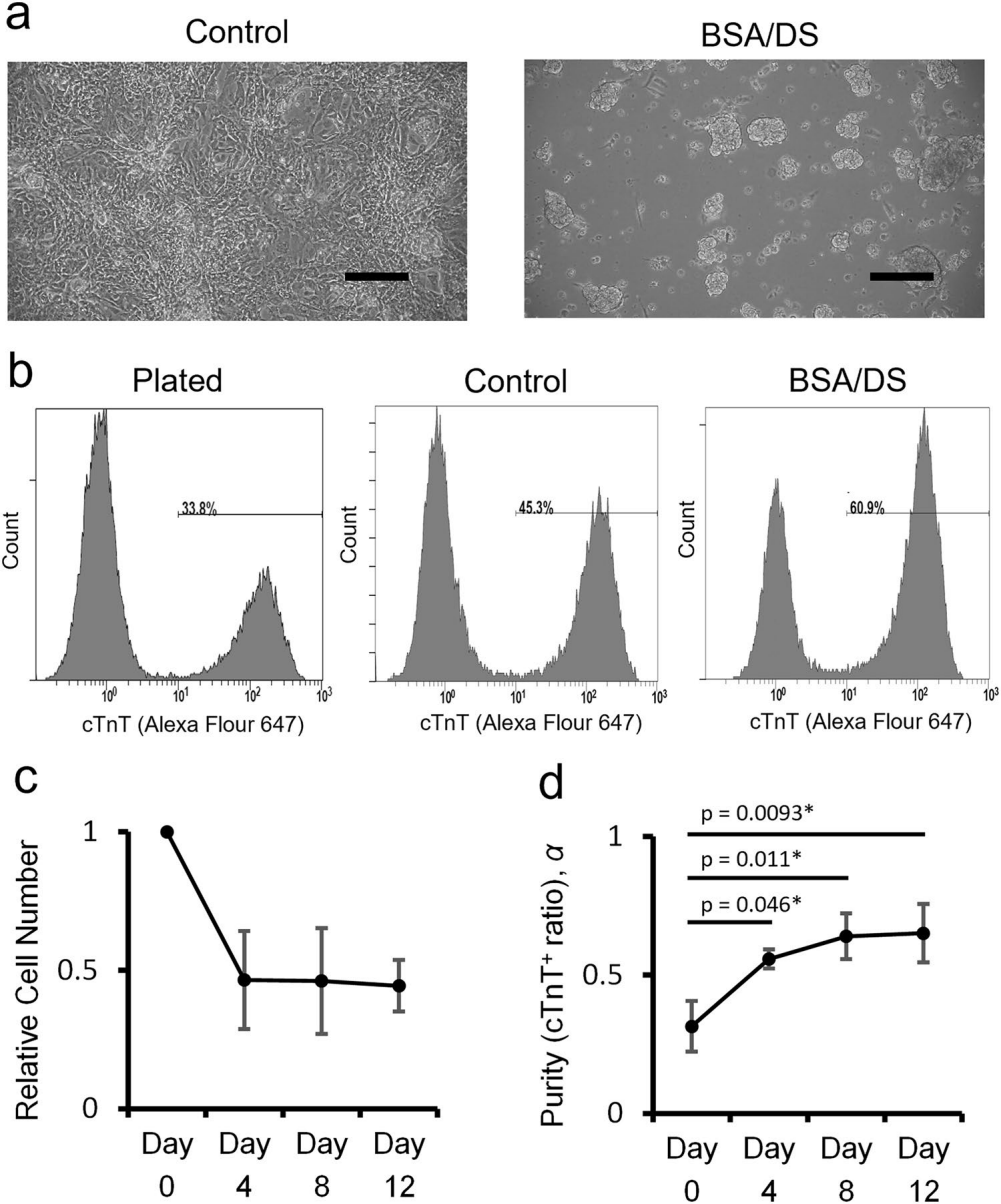
BSA/DS culture enriches cardiomyocytes. hiPSC-derived cardiomyocytes were cultured in serum-free medium with or without the addition of 1 mg/mL BSA and 1 mg/mL DS. (**a**) Representative phase contrast microscopic images of the cells from each group prior to harvest on day 4. Scale bars: 200 μm. (**b**) Representative histograms of flow cytometric analysis for cells on day 0 (plated) and day 4 (control and BSA/DS). (**c**) Relative total cell numbers. (**d**) cTnT-positive ratio. p-values: Dunnett’s test. N = 3. Error bars represent standard deviation. Asterisks indicate p < 0.05.

We previously reported that BSA and high molecular weight DS synergistically prevent cell adhesion in serum-free medium^13^ (referred to as BSA/DS culture). Initially, we examined whether the prevention of cell attachment by BSA/DS supplementation affects cardiomyocyte purity. hiPSC-derived cardiomyocytes were plated with or without BSA/DS in serum-free medium, and the harvested cells were analyzed by flow cytometry (Fig. 1). Consistent with our previous findings, the addition of BSA/DS prevented cell adhesion, resulting in the formation of aggregates (Fig. 1a). Furthermore, the cardiomyocyte purity appeared to be higher in the BSA/DS group compared to the control group on day 4 (Fig. 1b). Furthermore, cardiomyocytes were monitored over time, revealing a significant increase in cTnT-positive ratios with culture progression (Fig. 1c–d).

### Culture on low-attachment dishes also exhibited cardiomyocyte enrichment

**Figure 2:**
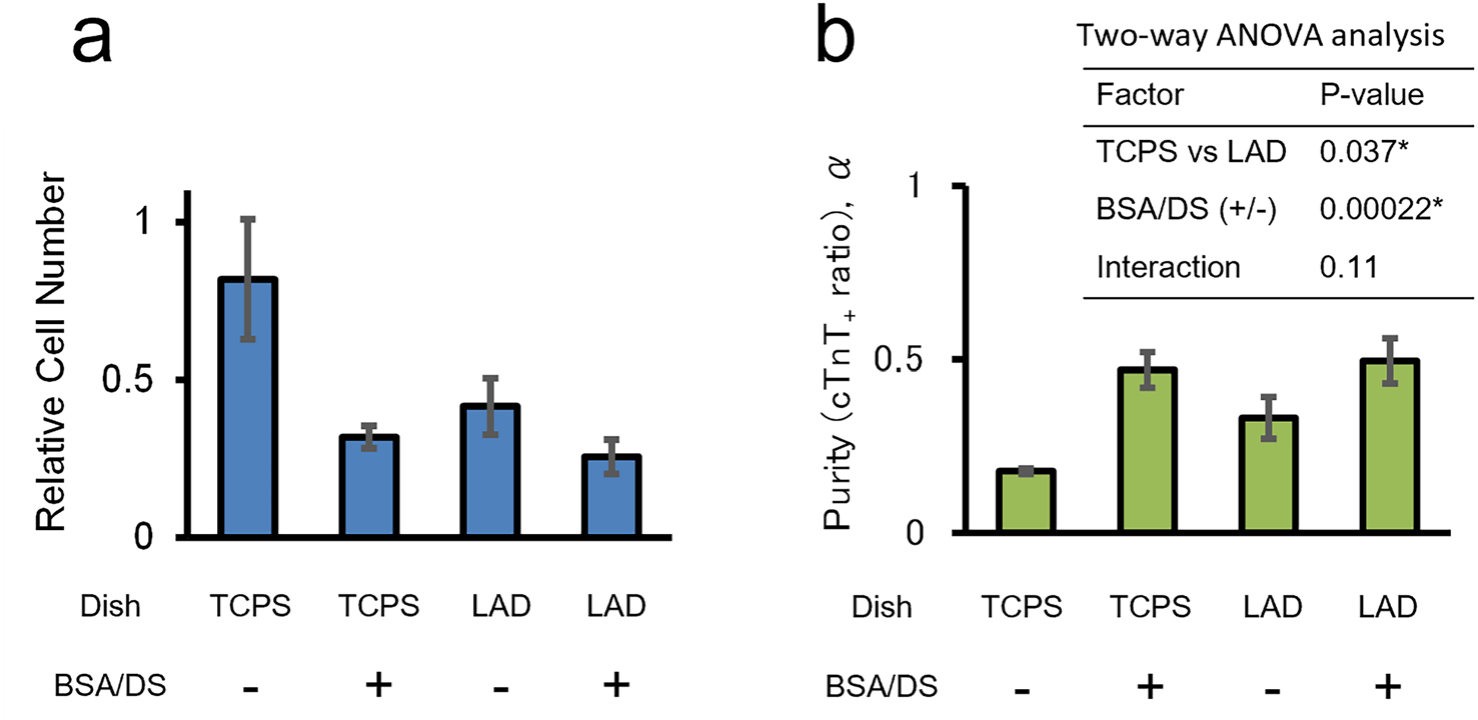
Low-attachment dish also enriches cardiomyocytes. hiPSC-derived cardiomyocytes were cultured in serum-free medium on tissue culture polystyrene dish (TCPS) or low-attachment dish (LAD) with or without the addition of 1 mg/mL BSA and 1 mg/mL DS. (BSA/DS). (**a**) Relative total cell numbers. (**b**) Analysis of cTnT-positive ratios with two-way ANOVA statistical test. N = 3. Error bars represent standard deviation. Asterisks indicate p < 0.05.

To investigate whether the increased population of cardiomyocytes under BSA/DS treatment is solely dependent on cell attachment or influenced by other mechanisms, commercially available low cell attachment dishes were tested. Following cardiac differentiation culture, the cells were plated in serum-free medium with or without BSA/DS using tissue culture dishes or low attachment dishes. After a 4 day culture period, the harvested cells were analyzed by flow cytometry (Fig. 2). The use of low cell attachment dishes also resulted in enrichment of cTnT-positive cardiomyocytes, suggesting that cardiomyocytes may exhibit a lower dependence on anchorage than other cell types, potentially indicating a degree of low-adhesion culture resistance.

### Repeated BSA/DS treatments increased purity but decreased yield

**Figure 3:**
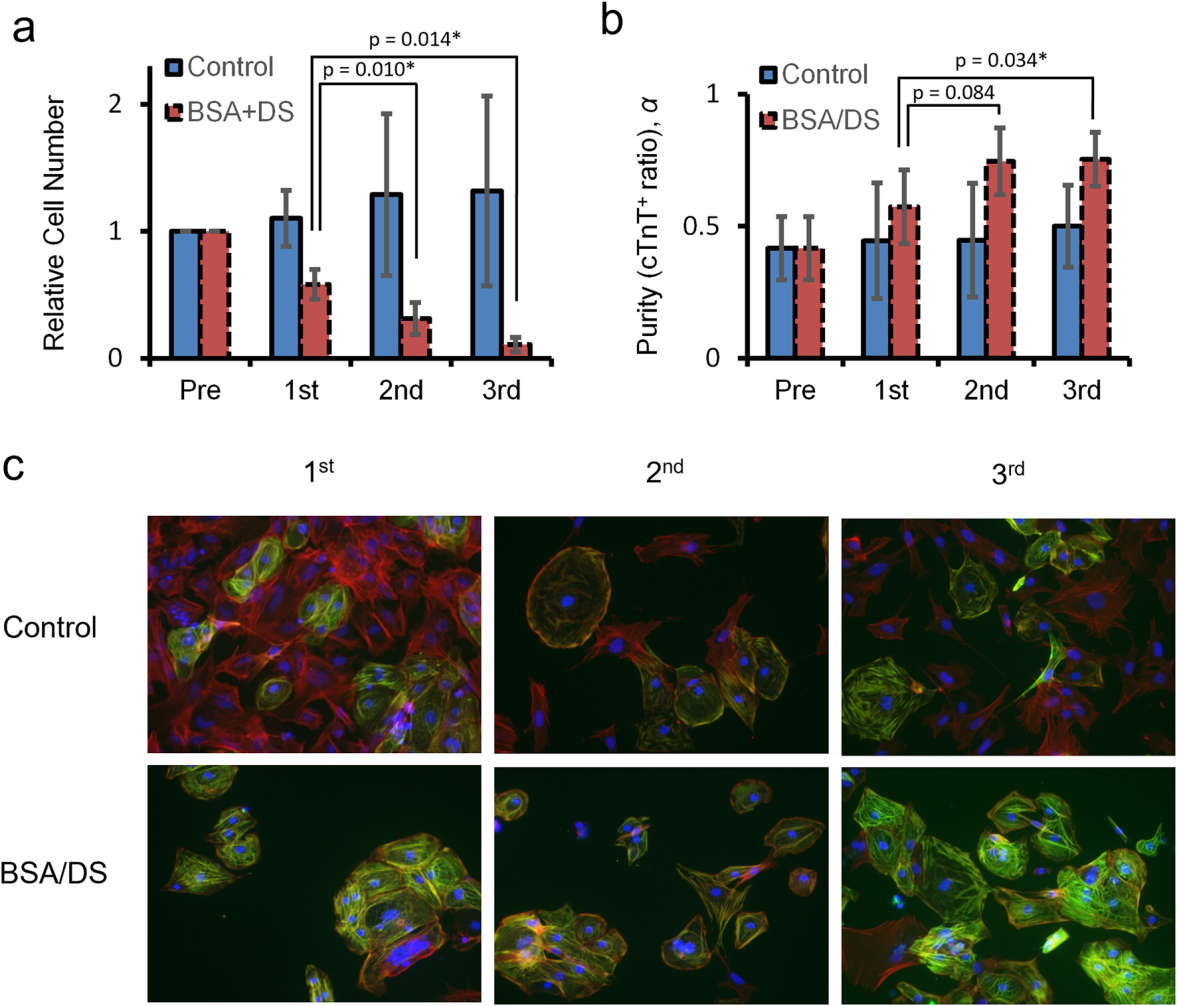
Repeated treatment with BSA/DS. hiPSC-derived cardiomyocytes were plated with or without 1 mg/mL BSA and 1 mg/mL DS. The cells were passaged every 4 days. (**a**) Relative total cell numbers. p-values: Dunnett’s test using the first round as the control. N = 3. Error bars represent standard deviation. Asterisks indicate p < 0.05. (**b**) cTnT-positive ratios. p-values: Dunnett’s test using the first round as the control. N = 3. Error bars represent standard deviation. Asterisks indicate p < 0.05. (**c**) Immunofluorescence of cells re-seeded in dishes without BSA/DS. Red: fibril actin (phalloidin). Green: cTnT. Blue: nuclei (DAPI).

To assess the practicality of BSA/DS culture as a selection method, we checked the effect of repeated treatments, involving cells passaging every 4 days under the same conditions (Fig. 3). The cTnT-positive ratio improved by repeated treatment (Fig. 3b) while the cell number dropped significantly over the passages (Fig. 3a).

### BSA/DS-treated cardiomyocytes kept basic function

**Figure 4:**
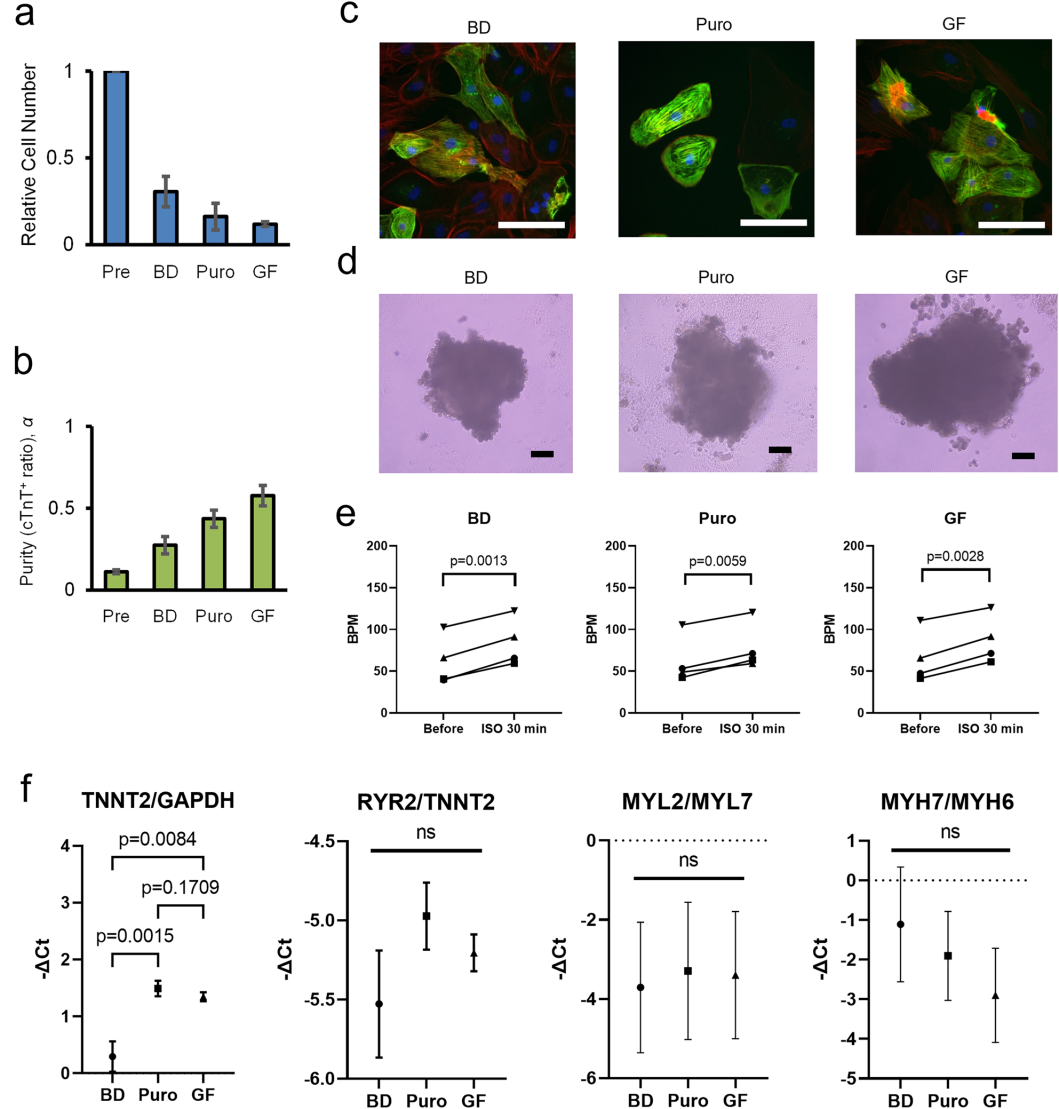
Functional analysis of treated cardiomyocytes. hiPSC-derive cardiomyocytes were treated by one of three selection methods: BSA/DS method (BD), puromycin-treatment (Puro), or glucose-free culture (GF). (**a**) Relative total cell numbers. (**b**) cTnT-positive ratio. (**c**) Immunofluorescence images of re-seeded cells. Blue: nucrei. Green: cTnT, Red: F-actin. Bar: 100 μm. (**d**) Microscopic images of the aggregates. Bar: 100 μm. (**e**) Beating rates of the aggregates before and 30 min after administering 100 nM isoproterenol (ISO). Each plot represents the average value of five aggregates for each experiment. N = 4. p-values: paired *t*-test. (**f**) qPCR analysis. N = 4. Error bars: SD. ns: p > 0.05 in one-way ANOVA. p-values: Tukey’s multiple comparison test.

Finally, we assessed the impact of BSA/DS treatment on cardiomyocyte function (Fig. 4). We compared hiPSC-derived cardiomyocytes treated with three selection methods: BSA/DS culture, puromycin treatment, or glucose-free culture. In terms of selection efficiency, the BSA/DS method seemed to have slightly weaker stringency compared with other two methods (Fig. 4a, b). Immunofluorescence showed the presence of cTnT-positive cardiomyocytes in all groups (Fig. 4c). To investigate the rhythmic function, cell aggregates were prepared by 5- or 6-day cultivation in low-adhesion U-bottom plates, and the beating rates were examined both before and after the addition of isoproterenol, a beta-stimulant (Fig. 4d, e). In all aggregate samples across all groups, autonomous beating was observed. The increase in beating rates following the addition of isoproterenol indicated that the cardiomyocytes remained functional even after treatments. To analyze maturation and subtype commitment, gene expression of the treated cardiomyocytes was examined using quantitative PCR (Fig. 4f). Concerning this analysis, it is important to note that a substantial number of non-cardiomyocytes are still present in the cell populations, as inferred from the difference in the relative expression levels of TNNT2 over an endogenous control (GAPDH). The relative expression levels of three cardiomyocyte-specific gene pairs, RYR2/TNNT2, MYL2/MYL7, and MYH7/MYH6, were compared as indicators of myocardial maturation and subtype commitment, revealing no significant differences.

## Discussion

In this study, we initially observed that employing a non-coating low-adhesion culture method using BSA/DS or commercially available low-adhesion dishes increased the proportion of cTnT-positive cells in hiPSC-derive cardiomyocytes (Fig. 1 and Fig. 2). This finding implies that cardiomyocytes may possess a higher resistance to low-adhesion culture compared to other cell types. Subsequently, we investigated whether this phenomenon could harness for cardiomyocyte selection. To optimize the processing conditions, we tried repeated treatments, but as expected, although the purity improved, the yield deteriorated (Fig. 3). Lastly, to evaluate the impact of treatments to function and phenotype of cardiomyocytes, we compared three selection methods: BSA/DS culture, puromycin-treatment, and glucose-free culture (Fig. 4). In all groups, the treated cardiomyocytes exhibited autonomous beating and beta-stimulant response. In addition, no significant difference was found in expression ratio of three cardiomyocyte-specific gene-pairs (RYR2/TNNT2, MYL2/MYL7 and MYH7/MYH6). In this comparison, BSA/DS method represented larger yield and smaller purity compared with other two method, suggesting relatively weak stringency.

There are several potential mechanisms underlying the selection of the cardiomyocytes in low-adhesion culture. One possibility is that detachment-related pathways, such as the YAP/TAZ pathway^14,15^, may differ between cardiomyocytes and other cells. Via these pathways, the failure of adhesion to the substrate triggers cell death. This could be a mechanism similar to anoikis. Another possibility is that specific cell–cell adhesion among cardiomyocytes could serve as a signal to evade cell death, as observed in non-transformed cells forming aggregates^16–18^. Additionally, the interplay between the glycolytic system and substrate adhesion warrants consideration. In cancer cells, detachment stress induces a metabolic switch from glycolysis to aerobic pathways^19–21^. The ability of cardiomyocytes to survive in glucose-free medium suggests their lesser reliance on glycolysis for energy. It’s conceivable that loss of substrate attachment could impair the glycolytic system, leading to a selection process akin to that observed in glucose-free conditions. In any case, this study did not offer sufficient insights into the exact mechanism at play.

Low-adhesion culture showed a relatively moderate selection stringency (Fig. 4a, b). This indicates a subset of non-cardiomyocytes may be resistant to low-adhesion culture. Previously, we established that the majority of non-cardiomyocytes are SM22-positive fibroblasts (mural cells), with only a small fraction being vWF-positive endothelial cells^22^. Gene expression analysis has revealed that these fibroblasts exhibit a profile more akin to cardiac fibroblasts than to dermal fibroblasts or mesenchymal stem cells^23^. In addition to the gene expression analysis targeting cardiomyocytes, we also examined marker genes for non-cardiomyocytes, albeit as a preliminary assessment (Supplementary Figure S2). In terms of relative expression to the endogenous control gene (GAPDH), there was no significant difference observed in COL1A1 (a fibroblast marker) and ACTA2 (a marker for smooth muscle cells or myofibroblasts), while CDH5 and PECAM1 (markers for endothelial cells) showed lower expression in the puromycin-treated group, suggesting that endothelial cells are more sensitive to puromycin selection. In other words, BSA/DS method is less effective in removing endothelial cells. Investigating the subpopulations within non-cardiomyocytes is an issue for future research.

Clinical researches are underway in the field of cardiac regenerative medicine utilizing pluripotent stem cell-derived cardiomyocytes, and the presence of a percentage of cTnT-negative cells in the cell population prepared for clinical studies was reported, underscoring the importance of purity control^24^. In the realm of regenerative medicine, preference is given to methods that minimize the likelihood of causing genomic alterations, such as those employing episomal vectors^25^. One commonly employed selection technique, which does not involve genetic modification, is the use of a glucose-free medium^10^. In this study, we present findings on a new selection method through low-adhesion culture. However, we were unable to demonstrate a clear advantage over the glucose-free medium in terms of performance. If we dare to mention a practical difference, while glucose-free medium is readily available in major synthetic media like DMEM and MEM, the BSA/DS method can be applied in any medium, requiring only the addition of BSA and DS. While the purities shown in Fig. 4b may not meet practical standards, these selection treatments can be applied repeatedly to improve purity. However, it is common for repeated treatments to diminish yields, as demonstrated in Fig. 3 for the BSA/DS method. To address this tradeoff between purity and yield, one approach is to combine two or more selection mechanisms. We tested the combination of glucose-free medium or puromycin treatment with BSA/DS culture (Supplementary Figure S1). While the combination with glucose-free medium reduced yield without enhancing purity, the combination with puromycin treatment did improve purity, albeit with potential yield reduction. These results might reflect the internal mechanism by which BSA/DS culture works. Another aspect in clinical use is the need to adequately remove tumorigenic undifferentiated cells. RT-PCR-based techniques have proven highly sensitive for detecting undifferentiated human iPS cells^26^. We examined the expression levels of the NANOG and LIN28 genes in the cells selected by each method (Supplementary Figure S2). Interestingly, we observed an absence of gene expression in cells selected by the BSA/DS culture method for LIN28, which is considered the most sensitive marker gene. This suggests that residual undifferentiated cells may be present at very low levels using the BSA/DS method. It is important to acknowledge a limitation of this study: since we employed a single cardiomyocyte differentiation method established in our laboratory, there may be variations in the selection outcomes when using alternative differentiation methods.

In conclusion, hiPSC-derived cardiomyocytes exhibited low-adhesion culture resistance compared to their accompanying non-contractile cells, and low-adhesion culture represents an alternative approach for selection of cardiomyocytes derived from pluripotent stem cells.

## Methods

### Reagents

The suppliers and catalog numbers of reagents used in this study are available in Supplementary Table S1 online.

### Cell culture media

The serum-free medium for cardiomyocyte selection was prepared by adding 0.01% ascorbic acid phosphate magnesium salt n-hydrate and 1% penicillin–streptomycin solution to Dulbecco’s Modified Eagle’s Medium (DMEM). The medium for glucose-free culture was prepared adding 4 mM sodium l-lactate, 0.01% ascorbic acid phosphate magnesium salt n-hydrate, and 1% penicillin–streptomycin solution to glucose-free DMEM.

### Preparation of cells

Cardiomyocytes were derived from hiPSC line 201B7 obtained from RIKEN (Tsukuba, Japan) using a previously reported method^22,27^. Initially, hiPSCs were genetically modified to express puromycin-resistance gene under the control of the α-myosin heavy chain promoter, enabling the subsequent selection of cardiomyocytes using puromycin. The hiPSCs were cultured on a feeder-layer of mitomycin C-treated mouse embryonic fibroblasts. Differentiation into cardiomyocytes was achieved using a designated suspended aggregation culture device. At the end of differentiation culture, the cells were seeded at a density of 1 × 10^7^ cell/dish in 100 mm tissue culture dishes containing DMEM supplemented with 10% fetal bovine serum (FBS) and 1% penicillin–streptomycin solution. The cells were incubated in a humidified 37 ℃ incubator with 5% CO_2_.

### Cardiomyocyte selection

The differentiated cells in tissue culture dishes were harvested using Trypsin–EDTA (37 ℃, 10 min), washed three times with serum-free DMEM, and then plated in 100 mm tissue culture dishes at a density of 3 × 10^6^ to 1 × 10^7^ cells/dish. Cardiomyocytes were treated with three main techniques. The first technique involved controlling cell attachment either by using media supplemented with BSA and high molecular weight DS (BSA/DS culture) or by using commercially available low cell attachment dishes (Corning Inc., C/N #3262). The second technique utilized glucose-free culture with lactate-supplemented glucose-free medium. The third technique involved puromycin treatment using the genetically introduced puromycin-resistance gene mentioned earlier. For puromycin treatment, 1.5 μg/mL puromycin dihydrochloride was added to the medium 24 h before harvest. Throughout the selection process, the medium was not replaced except during passaging.

### Cell count and flow cytometry

To determine cell numbers and cardiomyocyte purities, the cells, including floating non-attached cells, were harvested using Trypsin–EDTA (37 ℃, 10 min). Cell counting was performed using a disposable cell counter (Waken B Tech, Kyoto, Japan) with Trypan Blue dye to exclude dead cells. Based on the calculated cell numbers, a sample of 5 × 10^5^ cells or less was collected for flow cytometry analysis. If necessary, the remaining cells were plated in new dishes after three washes with serum-free DMEM. The sampled cells were immediately stained by LIVE/DEAD fixable green dead cell stain kit according to the manufacturer’s instructions (1 μL in 1 mL, 30 min, RT). Subsequently, the cells were centrifuged, resuspended in a 1% paraformaldehyde solution, and stored at 4 ℃. After 30 min or more, the cells were centrifuged and resuspended in phosphate buffered saline (PBS) containing 0.1% Triton X-100, 5% blocking one solution, and a 1/200 dilution of anti-cTnT antibody. After 30 min, the cells were centrifuged and resuspended in PBS with 0.1% Triton X-100, 5% blocking one solution, a 1/200 dilution of Alexa Fluor 647 anti-mouse IgG, and 2.5 μg/mL 4ʹ,6-diamidino-2-phenylindole (DAPI). Following another 30 min, the cells were centrifuged and resuspended in PBS. The cell suspension samples were analyzed using a Gallios flow cytometer (Beckman Coulter life sciences). The live cell population was gated using the fluorescence of DAPI and LIVE/DEAD fixable green dead cell stain kit. The ratio of cTnT-positive cells was determined using the fluorescence intensity threshold set at the geometric average between the low intensity peak position and the high intensity peak position.

### Analysis of cardiomyocyte selection efficiency

The relative cell number, *R*, for each selection procedure was determined by comparing the number of cells plated and harvested. The accumulated relative cell number for repeated procedures (such as passaging) was calculated by multiplying the *R* values of each step. Cardiomyocyte purity, *α*, in a cell suspension sample was determined using flow cytometry as described above.

### Immunofluorescence

The cells on the substrates were fixed using a paraformaldehyde aqueous solution. After rinsing with PBS, the cells were treated with a mouse monoclonal antibody for human cTnT. Subsequently, they were treated with Alexa Fluor 647 anti-mouse IgG secondary antibody, Alexa Fluor 568 conjugated phalloidin, and DAPI.

### Measuring beating rates

The cells were seeded in five wells of an EZ-BindShut SP non-adherent U-bottom 96-well plate (AGC Techno Glass) at 1 × 10^4^ cells/well in serum-free medium. After 5 or 6 days, microscopic videos (> 30 s) were captured both before and 30 min after the administration of 100 nM isoproterenol. Beating rates were measured from the captured videos.

### Gene expression analysis

The cells were cultured in a 3.5 cm tissue culture dish with serum-free medium for 5 days to allow recovery from treatment stresses. Total RNA was extracted using the RNeasy plus mini kit (Qiagen) according to the manufacturer’s instructions. cDNA was synthesized from the RNA using a RT-RamDA cDNA synthesis kit (Toyobo). Subsequently, gene expression was analyzed through TaqMan RT-PCR assay on a ViiA 7 real-time PCR system (Thermo Fisher Scientific). TaqMan probes for the target genes can be found in Supplementary Table S2 online. For comparison of the expression levels of each gene, we calculated − ΔCt as Ct^ref^–Ct^goi^ where Ct^ref^ is the Ct value of reference gene (GAPDH) and Ct^goi^ is the Ct value of the gene of interest. Higher − ΔCt indicates higher expression of the gene of interest. Additionally, we computed − ΔCt of three cardiomyocyte-specific gene pairs, RYR2/TNNT2, MYL2/MYL7, and MYH7/MYL6. In this case, the gene after the slash is the reference gene.

### Statistical analysis

Statistical analysis was conducted using paired Student’s *t*-test, two-way ANOVA, Dunnett’s test or one-way ANOVA with Greenhouse–Geisser correction. The results are presented on each figure.

### Supplementary Information

Supplementary Figure S1.

Supplementary Figure S2.

Supplementary Table S1.

Supplementary Table S2.

## Data Availability

All data that support the findings of this research are included within the article and the supplementary files.

## References

[1] Ando H (2017). A new paradigm for drug-induced torsadogenic risk assessment using human iPS cell-derived cardiomyocytes. J. Pharmacol. Toxicol. Methods.

[2] Nakamura Y (2014). Assessment of testing methods for drug-induced repolarization delay and arrhythmias in an iPS cell-derived cardiomyocyte sheet: Multi-site validation study. J. Pharmacol. Sci..

[3] Asakura K (2015). Improvement of acquisition and analysis methods in multi-electrode array experiments with iPS cell-derived cardiomyocytes. J. Pharmacol. Toxicol. Methods.

[4] Braam SR (2010). Prediction of drug-induced cardiotoxicity using human embryonic stem cell-derived cardiomyocytes. Stem Cell Res..

[5] Clements M, Millar V, Williams AS, Kalinka S (2015). Bridging functional and structural cardiotoxicity assays using human embryonic stem cell-derived cardiomyocytes for a more comprehensive risk assessment. Toxicol. Sci..

[6] Harris K (2013). Comparison of electrophysiological data from human-induced pluripotent stem cell-derived cardiomyocytes to functional preclinical safety assays. Toxicol. Sci..

[7] Qu Y, Vargas HM (2015). Proarrhythmia risk assessment in human induced pluripotent stem cell-derived cardiomyocytes using the maestro MEA platform. Toxicol. Sci..

[8] Zhao M, Tang Y, Zhou Y, Zhang J (2019). Deciphering role of Wnt signalling in cardiac mesoderm and cardiomyocyte differentiation from human iPSCs: Four-dimensional control of Wnt pathway for hiPSC-CMs differentiation. Sci. Rep..

[9] Friedman CE (2018). Single-cell transcriptomic analysis of cardiac differentiation from human PSCs reveals HOPX-dependent cardiomyocyte maturation. Cell Stem Cell.

[10] Tohyama S (2013). Distinct metabolic flow enables large-scale purification of mouse and human pluripotent stem cell-derived cardiomyocytes. Cell Stem Cell.

[11] Klug MG, Soonpaa MH, Koh GY, Field LJ (1996). Genetically selected cardiomyocytes from differentiating embryonic stem cells form stable intracardiac grafts. J. Clin. Invest..

[12] Kadari A (2015). Robust generation of cardiomyocytes from human iPS cells requires precise modulation of Bmp and Wnt signaling. Stem Cell Rev. Rep..

[13] Kikuchi T, Matsuura K, Shimizu T (2021). Non-coating method for non-adherent cell culture using high molecular weight dextran sulfate and bovine serum albumin. J. Biosci. Bioeng..

[14] Zhao B (2012). Cell detachment activates the Hippo pathway via cytoskeleton reorganization to induce anoikis. Genes Dev..

[15] Nakagawa H, Higurashi M, Ishikawa F, Mori K, Shibanuma M (2023). An indispensable role of TAZ in anoikis resistance promoted by OTUB1 deubiquitinating enzyme in basal-like triple-negative breast cancer cells. Biochem. Biophys. Res. Commun..

[16] Luebke-Wheeler JL, Nedredal G, Yee L, Amiot BP, Nyberg SL (2009). E-cadherin protects primary hepatocyte spheroids from cell death by a caspase-independent mechanism. Cell Transplant..

[17] Hofmann C (2007). Cell–cell contacts prevent anoikis in primary human colonic epithelial cells. Gastroenterology.

[18] Krawetz RJ, Li X, Rancourt DE (2009). Human embryonic stem cells: Caught between a ROCK inhibitor and a hard place. Bioessays.

[19] Uen W, Tseng T, Wu C-P, Lee S (2022). Detachment stress mediated bioenergetic switch of malignant melanoma cells into anti-Warburg phenotype. Aging.

[20] Schafer ZT (2009). Antioxidant and oncogene rescue of metabolic defects caused by loss of matrix attachment. Nature.

[21] Mason JA (2021). SGK1 signaling promotes glucose metabolism and survival in extracellular matrix detached cells. Cell Rep..

[22] Matsuura K (2012). Creation of human cardiac cell sheets using pluripotent stem cells. Biochem. Biophys. Res. Commun..

[23] Masuda S, Matsuura K, Shimizu T (2018). Inhibition of LYPD1 is critical for endothelial network formation in bioengineered tissue with human cardiac fibroblasts. Biomaterials.

[24] Miyagawa S (2022). Case report: Transplantation of human induced pluripotent stem cell-derived cardiomyocyte patches for ischemic cardiomyopathy. Front. Cardiovasc. Med..

[25] Ichimura H, Shiba Y (2017). Recent progress using pluripotent stem cells for cardiac regenerative therapy. Circ. J..

[26] Kuroda T (2012). Highly sensitive in vitro methods for detection of residual undifferentiated cells in retinal pigment epithelial cells derived from human iPS cells. PLoS One.

[27] Matsuura K (2016). TRPV-1-mediated elimination of residual iPS cells in bioengineered cardiac cell sheet tissues. Sci. Rep..

