# Supplementary material for: Low-adhesion culture selection for human iPS cell-derived cardiomyocytes
Source: Sci Rep. 2024 May 15;14:11081. doi: 10.1038/s41598-024-60765-5 (PMC11094004; doi:10.1038/s41598-024-60765-5)
Supplement: Supplementary file 1 — Supplementary Figure S1. [file 41598_2024_60765_MOESM1_ESM.docx]

**Supplementary Figure S1. Combination of conventional methods and BSA/DS.**

a) hiPSC-derived cardiomyocytes were cultured four days in glucose-free medium with or without BSA/DS. b) hiPSC-derived cardiomyocytes were cultured four days (in glucose-containing medium) with or without BSA/DS. At day 3, 1.5 μg/mL puromycin dihydrochloride was added to the medium. N = 3. Error bars: SD. P-values: paired t-test.
