# Supplementary material for: Low-adhesion culture selection for human iPS cell-derived cardiomyocytes
Source: Sci Rep. 2024 May 15;14:11081. doi: 10.1038/s41598-024-60765-5 (PMC11094004; doi:10.1038/s41598-024-60765-5)
Supplement: Supplementary file 2 — Supplementary Figure S2. [file 41598_2024_60765_MOESM2_ESM.docx]

**Supplementary Figure S2. Evaluation of residual non-cardiomyocytes.**

Relative expression levels against GAPDH for human induced pluripotent stem cell-derived cardiomyocytes treated by one of BSA/DS method (BD), puromycin method (Puro), or glucose-free method (GF). The selected cells were re-cultured for five days and then qPCR analysis was conducted for the cells. N = 4. Error bars: SD. n.d.: Not detected in any samples. ns: p > 0.05 in one-way ANOVA. P-values: paired t-test (for the comparison between two groups) or post-hoc Tukey’s multiple comparison test (for the comparison among three groups).
