# Supplementary material for: Low-adhesion culture selection for human iPS cell-derived cardiomyocytes
Source: Sci Rep. 2024 May 15;14:11081. doi: 10.1038/s41598-024-60765-5 (PMC11094004; doi:10.1038/s41598-024-60765-5)
Supplement: Supplementary file 3 — Supplementary Table S1. [file 41598_2024_60765_MOESM3_ESM.docx]

| Reagents | Source | Cat. No. / Product Name |
| --- | --- | --- |
| Dulbecco's Modified Eagle's Medium (DMEM) | Sigma-Aldrich | D6429 |
| Dulbecco's Modified Eagle's Medium, glucose-free | Thermo Fisher Scientific | 11966-025 |
| Fetal bovine serum (FBS) | Japan Bio Serum | - |
| Penicillin-streptomycin solution | Thermo Fisher Scientific | 15140-122 |
| Ascorbic acid phosphate magnesium salt n-hydrate | FUJIFILM Wako Pure Chemical Corporation | 013-12061 |
| 7.5% Bovine serum albumin (BSA) | Thermo Fisher Scientific | 15260-037 |
| Dextran sulfate sodium salt (DS) | Sigma-Aldrich | D8906 |
| Sodium L-lactate | Sigma-Aldrich | #71718 |
| Puromycin dihydrochloride | Thermo Fisher Scientific | A1113803 |
| Trypsin-EDTA (0.5w/v% Trypsin, 5.3mmol/L EDTA・4Na, x10) | FUJIFILM Wako Pure Chemical Corporation | 208-17251 |
| LIVE/DEAD Fixable Green Dead Cell Stain Kit | Thermo Fisher Scientific | L23101 |
| 0.5%-Trypan Blue Stain Solution | Nacalai tesque | 29853-34 |
| Triton X-100 | Sigma-Aldrich | T8787 |
| Blocking One Solution | Nacalai tesque | 05999-84 |
| Phosphate Buffered Saline (PBS) | Sigma-Aldrich | D1408 |
| Anti-cardiac troponin T antibody | Thermo Fisher Scientific | MS-295-P |
| Alexa Fluor 647 anti-mouse IgG | Thermo Fisher Scientific | A11001 |
| 4',6-diamidino-2-phenylindole (DAPI) | Thermo Fisher Scientific | D3571 |
| QuantAccuracy, RT-RamDA cDNA Synthesis Kit | TOYOBO | RMQ-101 |
| RNeasy Plus Mini Kit | QIAGEN | #74134 |
| TaqMan Fast Advanced Master Mix | Thermo Fisher Scientific | #4444556 |
| TaqMan Gene Expression Assay | Thermo Fisher Scientific | #4331182 |

**Supplementary Table S1. List of reagents.**

Providers and catalog numbers for reagents used in the study are listed.
