# Supplementary material for: Low-adhesion culture selection for human iPS cell-derived cardiomyocytes
Source: Sci Rep. 2024 May 15;14:11081. doi: 10.1038/s41598-024-60765-5 (PMC11094004; doi:10.1038/s41598-024-60765-5)
Supplement: Supplementary file 4 — Supplementary Table S2. [file 41598_2024_60765_MOESM4_ESM.docx]

| Target gene | Assay ID |
| --- | --- |
| GAPDH | Hs02758991_g1 |
| TNNT2 | Hs00165960_m1 |
| RYR2 | Hs00181461_m1 |
| MYL7 | Hs01085598_g1 |
| MYL2 | Hs00166405_m1 |
| MYH7 | Hs01110632_m1 |
| MYH6 | Hs01101425_m1 |
| LIN28 | Hs00702808_s1 |
| NANOG | Hs02387400_g1 |
| COL1A1 | Hs00164004_m1 |
| ACTA2 | Hs00426835_g1 |
| CDH5 | Hs00901465_m1 |
| PECAM1 | Hs00169777_m1 |

**Supplementary Table S2. TaqMan probes.**

TaqMan probes used for gene expression assay are listed.
